# Three-Dimensional Printing of Calcium Phosphate-Mesoporous Bioactive Glass Scaffolds for Bone Tissue Engineering

**DOI:** 10.3390/jfb16120463

**Published:** 2025-12-16

**Authors:** Ana Beatriz Gomes de Carvalho, Lais Medeiros Cardoso, Igor Paulino Mendes Soares, Joyce Rodrigues de Souza, Arpita Roy, Prabaha Sikder, Aldo R. Boccaccini, Eliandra de Sousa Trichês, Marco C. Bottino

**Affiliations:** 1 Department of Cariology, Restorative Sciences, and Endodontics, School of Dentistry, University of Michigan, Ann Arbor, MI 48109, USA; ana.b.carvalho@unesp.br (A.B.G.d.C.); lais.cardoso@unesp.br (L.M.C.); igor.soares@unesp.br (I.P.M.S.); joyce.souza@unesp.br (J.R.d.S.); arpitaro@umich.edu (A.R.); eliandra.sousa@unifesp.br (E.d.S.T.); 2 Department of Dental Materials and Prosthodontics, São Paulo State University, São José dos Campos 12245-000, SP, Brazil; 3 Department of Dental Materials and Prosthodontics, São Paulo State University, Araraquara 14801-385, SP, Brazil; 4 Department of Mechanical Engineering, Cleveland State University, Cleveland, OH 44115, USA; p.sikder@csuohio.edu; 5 Institute of Biomaterials, University of Erlangen-Nuremberg, 91085 Erlangen, Germany; aldo.boccaccini@fau.de; 6 Institute of Science and Technology, Federal University of São Paulo, São José dos Campos 12231-280, SP, Brazil; 7 Department of Biomedical Engineering, College of Engineering, University of Michigan, Ann Arbor, MI 48109, USA

**Keywords:** calcium phosphate cement, mesoporous bioactive glass, 3D printing, bone tissue engineering, osteogenic differentiation

## Abstract

Calcium phosphate cements (CPCs) and biomaterials, such as mesoporous bioactive glass (MBG), are critical for bone tissue engineering. This study aimed to 3D-print CPC scaffolds modified with MBG to enhance their osteogenic potential and regenerative ability. MBG powder was synthesized and characterized using transmission electron microscopy (TEM), X-ray diffraction (XRD), and nitrogen adsorption–desorption techniques. A commercial CPC ink (hydroxyapatite/α-tricalcium phosphate) was mixed with 5% MBG (*w*/*w*; CPC/MBG), and, after rheological assessment, the mixture was used to obtain scaffolds via 3D printing. These scaffolds were then tested for chemical, morphological, and mechanical properties, as well as ion release analysis. Unmodified CPC 3D-printed scaffolds served as controls. Biological experiments, including cell viability, DNA content, cell adhesion/spreading, and osteogenic gene expression, were performed by seeding alveolar bone-derived mesenchymal stem cells onto the scaffolds. Statistics were performed using Student’s *t*-test and ANOVA with post hoc tests (α = 5%). MBG characterization showed a typical mesoporous structure with aligned microchannels and an amorphous structure. Both formulations released calcium and phosphate ions; however, CPC/MBG also released silicon. Cell viability, adhesion/spreading, and DNA content were significantly greater in CPC/MBG scaffolds compared to CPC (*p* < 0.05) after 3 and 7 days of culture. Furthermore, CPC/MBG supported increased expression of key osteogenic genes, including collagen (COL1A1), osteocalcin (OCN), and Runt-related transcription factor 2 (RUNX2), after 14 days (*p* < 0.05). The combination of CPC ink with MBG particles effectively enhances the biocompatibility and osteogenic potential of the scaffold, making it an innovative bioceramic ink formulation for 3D printing personalized scaffolds for bone regeneration.

## 1. Introduction

A central challenge in bioengineering is developing biomaterials that replicate the properties of natural tissues. This challenge is especially complex in bone regeneration, as bone tissue involves not only intricate geometry but also unique molecular, chemical, and mechanical aspects [1,2]. Ideally, biomaterials for bone tissue engineering applications should provide sufficient mechanical strength while also exhibiting bioactivity to stimulate osteoblast differentiation and promote new bone formation [3,4,5].

Bioceramics are a crucial class of materials for bone regeneration and are available in various commercial forms such as grafts, scaffolds, filling materials, cements, and others [6,7]. Among these, calcium phosphate (CaP) bioceramics are the most widely used materials for orthopedic and craniomaxillofacial bone reconstructions [6,8] owing to their chemical resemblance to the mineral phase of bone, which ensures excellent biocompatibility [9]. A particularly valuable form of CaP is calcium phosphate cement (CPC), which consists of a liquid carrier phase and a powder precursor, such as α-tricalcium phosphate (α-TCP). When these components are mixed, dissolution and precipitation reactions occur, forming calcium-deficient hydroxyapatite (CDHA), which self-sets and hardens [10,11].

In recent years, advanced formulations of CPCs have led to the development of commercially available, ready-to-use injectable pastes, in which the setting process is delayed until the material is immersed in water. Biologically, CPCs possess key attributes, including high biocompatibility, biodegradability, and bone-forming properties [7,12]. Additionally, two important features make CPCs advantageous: (i) porosity, which enables the interaction of a drug delivery system, allowing CPC to be combined with bioparticles, proteins, and growth factors [10], and (ii) injectability and prolonged setting reaction, which expand the materials’ usability and facilitate scaffold fabrication [13].

To further enhance CPC’s bioactivity and osteogenic potential, its combination with other biomaterials [14,15], such as mesoporous bioactive glass (MBG), has emerged as a promising strategy [5,16]. MBGs are granular biomaterials that chemically bond with native bone [17]. Their mesoporous structure—with pore sizes ranging from 2 to 50 nanometers [18], modulated by the surfactant type used during synthesis [19,20]—greatly enhances their surface area. This architecture enables MBGs to act as effective carriers for therapeutic ions or biomolecules. In addition, MBGs can release ion products that stimulate osteoblastic differentiation [21]. When CPC and MBGs are combined to form scaffolds, the material exhibits greater microporosity and increased surface area. Additionally, MBG dissolution triggers ion release [8,22] which supports bone metabolism [8,23,24].

3D printing has become a widely used technique for scaffold fabrication [19,25,26], particularly extrusion-based methods such as direct ink writing (DIW). These approaches enable the precise, reproducible production of bioceramic scaffolds with customized macro- and microgeometries [7,27]. During the printing process, a pre-designed 3D model is built layer by layer through material deposition [19]. Achieving optimal printability and structural integrity requires that printing parameters align with the rheological properties of the ink [28], which depend on the composition and type of additives in the ink formulation [29].

Previous studies have demonstrated the successful printing of CPC [8,30], highlighting the potential for creating patient-specific scaffolds [29,31]. Although CPC/MBG [8,30,31,32] composites have been investigated, several key aspects remain underexplored, such as optimizing printing parameters for DIW to produce scaffolds with complex geometries [33] and determining the ideal MBG concentration to enhance the scaffolds’ biological and mechanical properties. Furthermore, the main difference between the present study and previous work is in the paste preparation method. CPC paste and MBG particles were manually mixed, resulting in a simpler formulation. In contrast, earlier studies used a more complex manual process in which CPC paste was combined with MBG particles and a carrier liquid (e.g., an aqueous Na_2_HPO_4_ solution) [30,34,35].

Considering this scenario, our study aimed to 3D-print CPC-based scaffolds modified with evaporation-induced self-assembly mesoporous bioactive glass (MBG) for bone tissue engineering. In this context, MBG was synthesized and characterized to confirm the successful formation of its mesoporous structure, and later the CPC/MBG mixture was used to produce 3D printed scaffolds. After fabrication, the scaffolds were subjected to chemical characterization, microstructural evaluation, mechanical testing, and ion release analysis. To validate their osteogenic potential, a series of biological assays (e.g., cell viability, DNA content, cell adhesion/spreading, and osteogenic marker gene expression) was performed using alveolar bone-derived mesenchymal stem cells onto the scaffolds. Overall, this work aims to provide a more straightforward approach to advancing the application of MBG-modified CPC pastes for bone regeneration. The proposed null hypotheses are as follows: (i) MBG modification does not influence CPC ink printability and scaffold fabrication, and (ii) the presence of MBG does not affect cell function and osteogenic potential.

## 2. Materials and Methods

### 2.1. MBG Preparation

A mesoporous bioactive glass (MBG) powder with a SiO_2_-CaO-P_2_O_5_ composition (80:15:5, mol%) was synthesized by evaporation-induced self-assembly (EISA) process following a previously established protocol [17]. The reagents were obtained from Sigma-Aldrich (St. Louis, MO, USA) and used as-received. Firstly, 4.0 g of Pluronic P123 (triblock copolymer of polyethylene oxide (PEO) and polypropylene oxide (PPO), PEO20-PPO70-PEO20, molecular weight M_W_ 5800) was dissolved in 60 g of ethanol (96%) with 1.0 g of 0.5 mol/L HNO_3_ solution and mixed at room temperature (RT) for 60 min. Next, 6.7 g of tetraethyl orthosilicate (TEOS, 99%), 0.73 g of triethyl phosphate (TEP, 99.8%), and 1.4 g of calcium nitrate-tetrahydrate (Ca(NO_3_)_2_ 4H_2_O) were added sequentially at three-hour intervals under continuous stirring. The solution was stirred at RT for 24 h, then cast onto Petri dishes and left to dry at RT for 1 day to undergo the EISA. The resulting gel (homogeneous and transparent membranes) was dried at 60 °C for 3 h and later calcined at 700 °C for 7 h. The final MBG powder was manually ground and sieved to obtain particles less than 45 μm. After synthesis, the MBG powder was characterized by transmission electron microscopy (TEM; Thermo Fisher Scientific, Waltham, MA, USA; Talos F200X G2 S/TEM, 200 kV) to evaluate the particles’ morphology and internal structure. X-ray diffraction (XRD; Rigaku Ultima IV diffractometer equipped with a CuKα source, Rigaku Americas Corporation, Woodlands, TX, USA) was performed at 40 kV and 20 mA to analyze the crystallographic structure. Nitrogen adsorption–desorption analysis was performed at 77 K using an Accelerated Surface Area and Porosimetry System (ASAP 2020, Micromeritics Instrument Corp., Norcross, GA, USA) to determine the textural properties. For this, the samples were dried under vacuum for 24 h at 105 °C. The specific surface area (S_BET_) was calculated using the Brunauer–Emmett–Teller (BET) method.

### 2.2. Rheological Analysis of CPC and CPC/MBG Pastes

Rheological measurements of CPC and CPC/MBG pastes were accomplished using a parallel-plate rheometer (TA Instruments, New Castle, DE, USA). Shear thinning feature and the viscosity of the pastes were measured by increasing the shear rate from 0.1 to 150 s^−1^ at 25 °C (distance between the plates was set at 100 μm) (*n* = 3).

### 2.3. CPC and CPC/MBG 3D Printed Scaffolds

**Figure 1:**
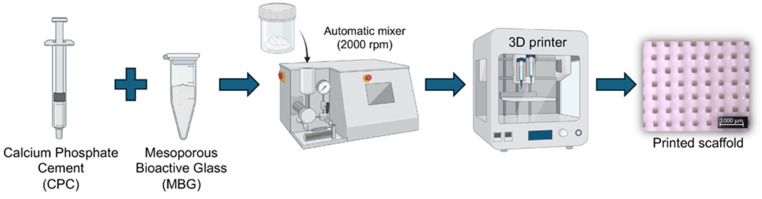
Schematic illustration of the 3D printing process for CPC and CPC/MBG scaffold fabrication.

Calcium phosphate cement (Hydroxyapatite/α-TCP; Plotter-Paste-CPC Innotere GmbH, Radebeul, Germany) was mixed with 5 wt.% of MBG (CPC/MBG) (*w*/*w*) using an automatic rotator (Flacketek, Inc., Dae 150.1 FVZ-K. Landrum, SC, USA) at 2000 rpm for 1 min. Then, the 3D printer cartridges were filled using a metal spatula and taken to the printer (3DDiscovery, RegenHU, Villaz-St-Pierre, Switzerland). Cylindrical scaffolds (5 mm × 3 mm) were designed using BioCAD software (RegenHU, Villaz-St-Pierre, Switzerland), and the generated G-code was exported to HMI CAM software (RegenHU, Villaz-St-Pierre, Switzerland) for printing the designed structures using a 25G plastic needle, a printing speed of 4 mm/s, a density of 40%, and a temperature of 25 ± 2 °C. Of note, unmodified CPC scaffolds were also printed and considered as the control group (CPC). All scaffolds were printed on a microscope glass slide, and after this, they were stored in distilled water at 37 °C for 72 h to set. A schematic diagram of the scaffolds’ production is shown in Figure 1.

### 2.4. Three-Dimensional Printed Scaffolds—Chemical Composition and Morphology

Fourier transform infrared spectroscopy (FTIR, Nicolet iS50, Thermo Fisher Scientific, Inc.) was run to assess the chemical functional groups of the scaffolds. For that, the samples (*n* = 3) were ground, and the powder was analyzed. Sixteen scans were collected over the 4000−500 cm^−1^ range with a resolution of 4, and the baseline-corrected spectra were normalized. XRD analysis was performed to verify the crystalline phases of the scaffolds using a Rigaku Ultima IV diffractometer with Cu Kα radiation (*λ* = 1.54 Å), over a 2θ range of 10–60°, with a step size of 0.05° and a scan speed of 1°/min.

Printed scaffolds were dried in a drying oven at room temperature for 72 h. The samples (*n* = 3) were gold-sputtered with a gold layer (~10 µm) to analyze their macro- and microstructure using field-emission scanning electron microscopy (FEG/SEM, Tescan MIRA3 FEG-SEM, Tescan USA Inc., Warrendale, PA, USA). Additionally, the presence of calcium (Ca), phosphorus (P), and silicon (Si) was mapped using energy-dispersive spectroscopy (EDS).

### 2.5. Ion Release Profile

The release of Ca^2+^, PO_4_^3−^, and Si^4+^ ions from the printed scaffolds (*n* = 4) was evaluated by inductively coupled plasma mass spectrometry (ICP-MS). The scaffolds were immersed in 1 mL of ultrapure water at 37 °C, and the medium was collected and renewed after 1, 3, and 7 days. After vortexing, the supernatants were filtered (0.22 µm), diluted (1:10,000), and analyzed using a NexION 300D ICP-MS system with Syngistix 2.2 software (PerkinElmer, Waltham, MA, USA). Ion quantification was performed using calibration curves from standard solutions, with yttrium as the internal standard. Measurements were carried out in six replicates, and results were expressed as ppm ± 95% confidence interval.

### 2.6. Mechanical Testing

Cylindrical-shaped scaffolds (8 mm × 3 mm; *n* = 4) were used to analyze compressive stress and compressive modulus of the scaffolds, comprising the mechanical properties. The tests were conducted using a universal testing machine (MTESTQuattro, ADMET Inc., Norwood, MA, USA) at a strain rate of 1 mm/min until 25–30% scaffold deformation. The elastic modulus was calculated from the stress–strain curve.

### 2.7. Alveolar Bone-Derived Mesenchymal Stem Cell—Scaffold Interaction

To evaluate the biological properties of the scaffolds, we used alveolar bone-derived mesenchymal stem cells (aBMSCs) kindly provided by Dr. Darnell Kaigler from the University of Michigan, School of Dentistry. The cells were isolated and characterized by their expression of surface markers CD73+, CD90+, and CD105+ [36]. In a 75 cm^2^ flask (Corning, New York, NY, USA), aBMSCs were cultured in complete alpha minimum essential medium (α-MEM, supplemented with Ribonucleosides, Deoxyribonucleosides, and L-Glutamine), 15% fetal bovine serum (FBS), and 1% antibiotic solution (Penicillin-Streptomycin 10,000 U/mL), all obtained from Gibco (Carlsbad, CA, USA). Cultures were maintained at 37 °C in an incubator with 5% CO_2_ (Thermo Fisher Scientific, Waltham, MA, USA) until reaching 75–80% confluence. Subculturing was performed using 0.25% trypsin-EDTA (Gibco), and cells from passages 3 to 7 were used for the experiments.

#### 2.7.1. Cell Viability

To assess the potential cell toxicity of 3D printed CPC and CPC/MBG scaffolds, cell viability and DNA content assays were performed. The scaffolds were first disinfected under ultraviolet light for 1 h on each side, placed in 24-well ultra-low attachment plates (Costar, Corning, New York, NY, USA), and then further dipped in 1 mL of 70% ethanol for 10 min. They were then rinsed twice with 1 mL of sterile phosphate-buffered saline (PBS; Gibco) for 5 min each. Afterward, 1 mL of complete α-MEM medium was added for 30 min, then removed. Finally, 5 × 10^4^ cells were seeded onto each scaffold, and 1 mL of α-MEM was added to each well 1 h after initial cell attachment.

Using the alamarBlue^®^ assay (Invitrogen, Carlsbad, CA, USA), the cell viability was evaluated after 1, 3, and 7 days of culture. In this assay, viable cells reduce resazurin to resorufin, resulting in a noticeable color change that is directly correlated to mitochondrial activity. At each time-point, cells were incubated for 3 h at 37 °C and 5% CO_2_ with a 10% alamarBlue^®^ solution (*v*/*v*) according to the manufacturer’s instructions. Fluorescence intensity was then measured using a spectrophotometer (SpectraMax iD3, Molecular Devices LLC, San Jose, CA, USA). By converting fluorescence values, cell viability was expressed as a percentage (%), with the control group (CPC) on day 1 set as 100%.

#### 2.7.2. DNA Content

The DNA content assay was performed using the Quant-iT PicoGreen dsDNA Assay Kit (Thermo Fisher Scientific, Waltham, MA, USA). This protocol is based on a fluorescent dye that specifically binds to double-stranded DNA (dsDNA), thereby providing an indirect measurement of cell proliferation. The fluorescence intensity, proportional to the dsDNA concentration in the sample, was measured using a spectrophotometer (SpectraMax iD3). After 1, 3, and 7 days of cell culture, the scaffolds were collected and transferred to tubes containing 500 mL of lysis buffer (10 mM Tris, 1 mM EDTA, and 0.2% *v*/*v* Triton X-100), followed by vortexing every 5 min for 30 min, and then kept on ice throughout the procedure [37]. Then, 100 µL of the supernatant was mixed with the Quant-iT PicoGreen dsDNA reagent, according to the manufacturer’s instructions. After 5 min, the fluorescence intensity was detected at excitation and emission wavelengths of 480 nm and 520 nm, respectively. The DNA content (ng/mL) was calculated based on a calibration curve constructed with lambda DNA standard.

#### 2.7.3. Cell Morphology, Adhesion, and Spreading

Following standard disinfection protocols, 5 × 10^4^ aBMSCs were seeded onto the scaffolds for 1, 3, and 7 days to analyze cell adhesion and spreading. After each time-point, the samples were rinsed with PBS and fixed in 4% paraformaldehyde for 30 min at RT, followed by dehydration with ascending ethanol concentrations (70%, 80%, 90%, and 100%, 15 min each). Subsequently, the samples were immersed in hexamethyldisilazane (HMDS, Sigma-Aldrich) overnight, later gold-sputtered and qualitatively analyzed by SEM (TESCAN MIRA3 FEG-SEM, Brun, Czech Republic) at 1000× (with a scale bar of 50 µm) and 5000× (with a scale bar of 10 µm) magnifications [38].

#### 2.7.4. Osteogenic Markers Gene Expression

Sterile 3D-printed CPC and CPC/MBG scaffolds were seeded with 1 × 10^5^ aBMSCs and cultured for 7, 14, and 21 days for qPCR evaluation of osteogenic gene expression [38]. Cells were lysed using TRIzol Reagent (Invitrogen). Total RNA was extracted using commercial kits, and cDNA was synthesized by reverse transcription according to the manufacturer’s protocols (Invitrogen). Real-time qPCR was then performed with standard reagents (TaqMan Gene Expression Master Mix; Thermo Fisher Scientific) to measure the expression levels of the target markers, including alkaline phosphatase (*ALPL*, Hs01029144_m1), collagen alpha 1 (*COL1A1*, Hs00164004_m1), osteocalcin (*OCN*; Hs01587814_g1), and runt-related transcription factor 2 (*RUNX2*; Hs01047973_m1). Gene expression levels were normalized to the housekeeping gene Glyceraldehyde 3-phosphate dehydrogenase (*GAPDH*; Hs02758991_g1). The relative gene expression levels of all genes (fold change relative to the CPC control group) were calculated using the 2^−ΔΔCT^ method.

### 2.8. Statistical Analysis

Statistics were performed using GraphPad Prism 10.0 (GraphPad Software, Inc., San Diego, CA, USA). Data related to mechanical testing, cell viability, DNA content, and osteogenic markers gene expression were assessed for distribution (Shapiro–Wilk test) and homogeneity of variances (F-test). Since the data adhered to a normal distribution, parametric statistical analyses were conducted using Student’s *t*-test (for mechanical testing and osteogenic markers gene expression) and two-way ANOVA with Sidak’s post hoc test (for cell viability and DNA content). The analysis of ion release data was conducted using confidence intervals. For all analyses, a pre-established 5% significance level was applied. Observations of cell adhesion and spreading from SEM were qualitatively described.

## 3. Results and Discussion

The present study employed material-based extrusion to fabricate 3D-printed scaffolds from CPC ink modified with MBG particles. This additive manufacturing technique is widely used to create organized, grid-like structures [7,27], ideal for bone regeneration applications [39]. Incorporating 5% MBG into CPC scaffolds enhanced their biological performance without compromising printability or geometric accuracy, thereby rejecting both null hypotheses.

Despite previous studies having examined CPC/MBG composites with varying MBG concentrations [8,30,31,32], our work is the first to comprehensively evaluate the optimal parameters for DIW while assessing both the mechanical and biological effects of CPC scaffolds containing 5% MBG. These findings demonstrate an effective strategy to improve scaffold biocompatibility and osteogenic potential, offering a promising approach to fabricate personalized scaffolds for bone regeneration.

### 3.1. MBG Powder Characterization

**Figure 2:**
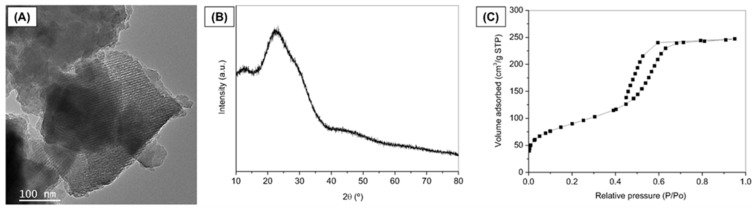
Characterization of MBG powder. (**A**) TEM image, (**B**) XRD pattern, and (**C**) Nitrogen adsorption/desorption isotherm.

MBG was successfully synthesized using the EISA method, and Figure 2 represents the characterization of the synthesized MBG powder. Representative TEM image reveals fringes appearing as parallel lines, indicating the formation of ordered mesoporous textures in the MBG (Figure 2A). XRD pattern confirmed the expected amorphous nature typical of bioactive glasses [38] (Figure 2B). The broad diffusion halo observed in the 2θ range of 15° to 35° of the diffractogram, without any intense diffraction peaks, confirms the amorphous nature of the prepared glass. The MBG isotherm is identified as a typical Type IV isotherm, characteristic of mesoporous solids (Figure 2C), according to the IUPAC classification.

Additionally, the adsorption–desorption curve within the mesopore range (P/P_0_ = 0.4 to 0.7) exhibits an H1-type hysteresis loop, indicating the presence of open-ended cylindrical mesopores with a narrow pore size distribution [40]. The MBG demonstrates a high specific surface area (313.4 m^2^/g), where values exceeding 100 m^2^/g are characteristic of mesoporous or mesostructured materials [40,41]. These results align with the findings of Anand et al. (2024) [42] and Sánchez-Salcedo et al. (2023) [43], who also synthesized MBGs by the same method described herein (EISA).

### 3.2. Rheological Behavior of CPC and CPC/MBG Paste

**Figure 3:**
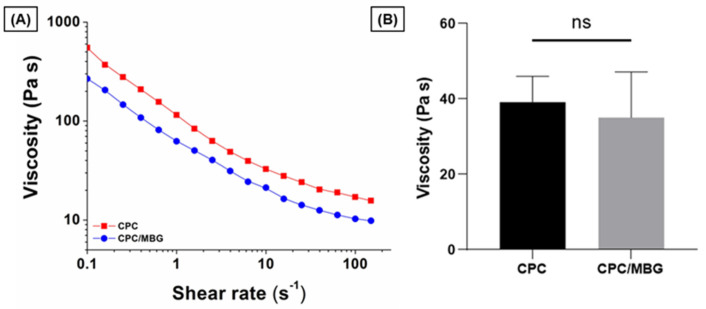
Flow curves of CPC and CPC/MBG pastes formulated for 3D-printed scaffold fabrication. (**A**) Representative viscosity plots of CPC and CPC/MBG at increasing shear rates from 0.1 to 150 s^−1^, (**B**) Measured viscosity of CPC and CPC/MBG at a shear rate of 10 s^−1^ (mean ± SD, *n* = 3). “ns” means not significant.

To assess the suitability of the pastes for 3D printing, their rheological properties were evaluated by increasing the shear rate from 0.1 to 150 s^−1^. The results showed that both paste formulations exhibited shear-thinning behavior, a key rheological property required for extrusion-based printing (Figure 3A). For comparison, the viscosity of the pastes was measured at a shear rate of 10 s^−1^, which, based on the reported literature, is appropriate for assessing the plotting behavior of these materials [44,45]. CPC (39.1 ± 6.8 Pa·s) and CPC/MBG (35.1 ± 12.0 Pa·s) displayed comparable viscosities with no statistically significant difference (Figure 3B), indicating both formulations possess suitable rheological characteristics for extrusion-based 3D printing.

### 3.3. Chemical and Morphological Characterization of 3D Printed Scaffolds

**Figure 4:**
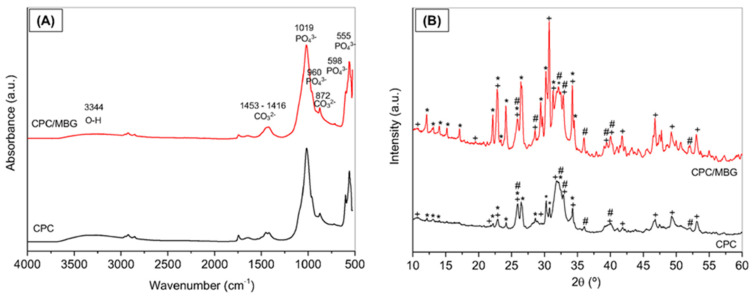
Characterization of the 3D-printed CPC and CPC/MBG scaffolds. (**A**) FTIR spectra and (**B**) XRD patterns. Legend: + Hydroxyapatite, Ca_5_(PO_4_)_3_(OH), * a-tricalcium phosphate (β-Ca_3_(PO_4_)_2_); and # carbonated hydroxyapatite, Ca_10_(PO_4_)_3_(CO_3_)_3_(OH)_2._

The FTIR spectra of CPC and CPC/MBG after 3 days of setting are shown in Figure 4A, which demonstrate the incorporation of mesoporous glass into the cement by an increase in band intensity and broadening in the Si-O-Si stretching region [46]. The vibration bands of the phosphate group (PO_4_^3−^) were mainly at the positions of 1019 cm^−1^ (asymmetrical stretching of P-O), 960 cm^−1^ (symmetrical stretching of P-O), 598, and 555 cm^−1^ (bending in the plane); a broad band around 3344 cm^−1^ is related to O-H group present in the hydroxyapatite (HA) structure; the bands at 1453-1416 cm^−1^ and 872 cm^−1^ were associated with the carbonate group (CO_3_^2−^), indicating that presence of carbonated hydroxyapatite (CHA). The infrared spectrum of CPC/MBG was very similar; however, a slight increase in band intensity and broadening in the Si-O-Si stretching region (1000–1100 cm^−1^) were observed, along with a shoulder at 950 cm^−1^, which is associated with the presence of silanol groups (Si-OH).

Figure 4B shows the XDR patterns of CPC and CPC/MBG. CPC scaffolds exhibit peaks corresponding to hydroxyapatite (HA, JCPDS #09-0432), carbonated hydroxyapatite (CHA, JCPDS #19-0272), and alpha-tricalcium phosphate (α-TCP, JCPDS #09-0348). CPC/MBG scaffolds displayed the same crystalline phases as CPC; however, the peaks associated with α-TCP were more pronounced, probably due to the release of silicon ions from the MBG, which may retard the hydration of α-TCP and subsequently hinder its conversion to hydroxyapatite. Conversely, Ca^2+^ and PO_4_^3−^ ions, originating from both CPC and MBG, can promote HA formation by increasing local supersaturation, thereby favoring nucleation and crystal growth [30].

**Figure 5:**
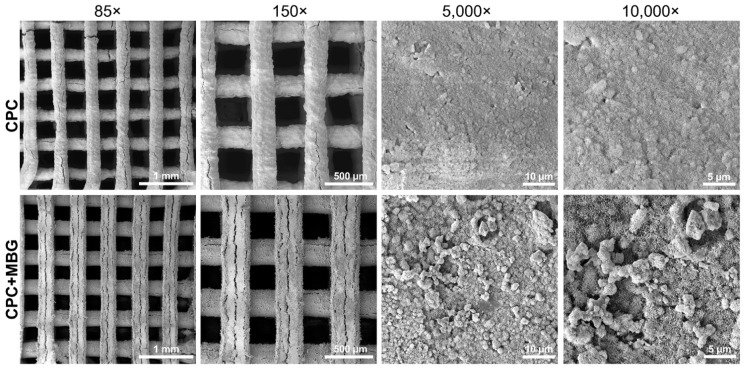
SEM images of the 3D-printed CPC and CPC/MBG scaffolds.

An adequate architecture was observed in SEM images (Figure 5), indicating a controlled 3D printing process, even with the addition of MBG. When printing different materials, adjusting the parameters is crucial because the ink’s composition can affect the flowability of the paste through the printer needle and, as a result, impact the printability of the scaffolds [47,48]. At lower magnifications (85× and 150×), both groups exhibited well-defined, homogeneous, and uniform strands. At higher magnifications (5000× and 10,000×), microstructural differences became evident. The CPC scaffolds exhibited a relatively smooth surface, whereas the CPC/MBG scaffolds showed a rougher texture with nanoscale features. Cracks appeared in both scaffolds and may be due to the SEM vacuum or the immersion setting procedure [13].

**Figure 6:**
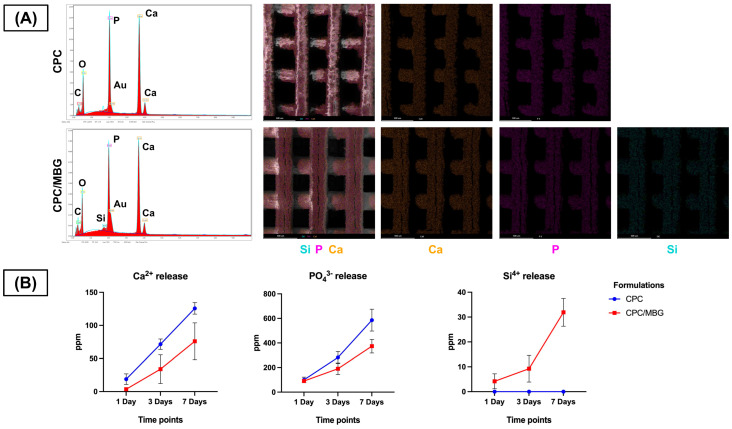
(**A**) EDS spectra and EDS/SEM elemental mapping of the 3D-printed CPC and CPC/MBG scaffolds. (**B**) Release profile of calcium, phosphate, and silicon ions (ppm) measured by ICP-MS from the scaffolds in ultrapure water after 1, 3, and 7 days. Points represent mean values, and error bars represent 95% confidence intervals (*n* = 6).

Meanwhile, EDS analysis ensured the presence of calcium (Ca) and phosphorus (P) in both scaffold formulations, confirming their calcium and phosphate composition (Figure 6A). Notably, silicon (Si) was exclusively detected in the CPC/MBG scaffolds, evidencing that the incorporation of MBG particles into the CPC matrix was effective. EDS/SEM elemental mapping further revealed a homogeneous distribution of Si throughout the CPC/MBG scaffolds, suggesting that the MBG particles were well integrated into the 3D-printed structure.

The ion release profile (Figure 6B) showed that both scaffolds released Ca^2+^ and PO_4_^3−^ ions over time, with CPC scaffolds exhibiting a slightly increased release compared to CPC/MBG. Additionally, Si^4+^ release was exclusively observed in the CPC/MBG scaffolds, displaying a sustained and gradual release over the 7-day period.

### 3.4. Mechanical Characterization

**Figure 7:**
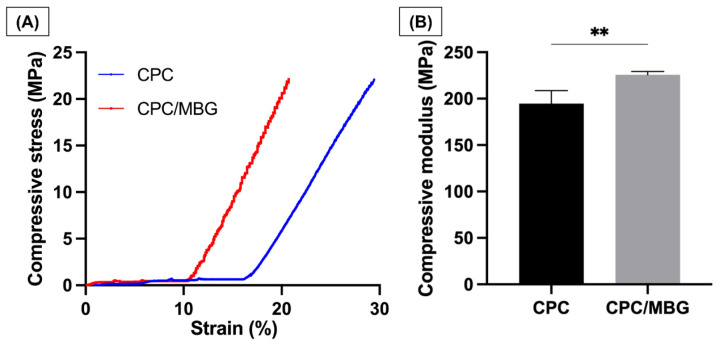
Mechanical properties. (**A**) Compressive stress, (**B**) Compressive modulus of elasticity. The results are presented as mean ± SD (*n*  =  4). Student’s *t*-test, ** = 0.0050.

Uniaxial compressive tests were performed to analyze the mechanical properties of the printed scaffolds. The stress–strain curves (Figure 7A) revealed that both CPC and CPC/MBG exhibited linear-elastic deformation, which agrees with the study of Richter et al., 2019 [30]. CPC exhibited a delayed onset of stress increase and continued to deform to a greater extent before reaching maximum stress. In contrast, CPC/MBG showed an earlier rise in stress and reached its peak at a lower strain than CPC. The compressive modulus findings (Figure 7B) showed that CPC/MBG (E = 225.8 MPa, SD = 3.50) had a significantly higher elastic modulus compared to CPC (E = 194.8 MPa, SD = 13.91), indicating that the presence of MBG particles improves the mechanical properties of CPC, thereby increasing its compressive strength. These results align with the findings of Luo et al. 2012 [49], who reported that 3D-printed alginate scaffolds with MBG showed an increase in compressive modulus with higher MBG content. An independent sample *t*-test confirmed a statistically significant difference, indicating that CPC/MBG has a greater capacity to withstand compressive deformation.

Although compressive strength analysis is widely used to evaluate the mechanical performance of CPC scaffolds [30,35], other mechanical parameters, such as fatigue strength, fracture toughness, and elastic energy absorption, are also important for assessing the suitability of scaffolds for bone tissue engineering and represent a limitation of the present study.

### 3.5. Biological Properties

**Figure 8:**
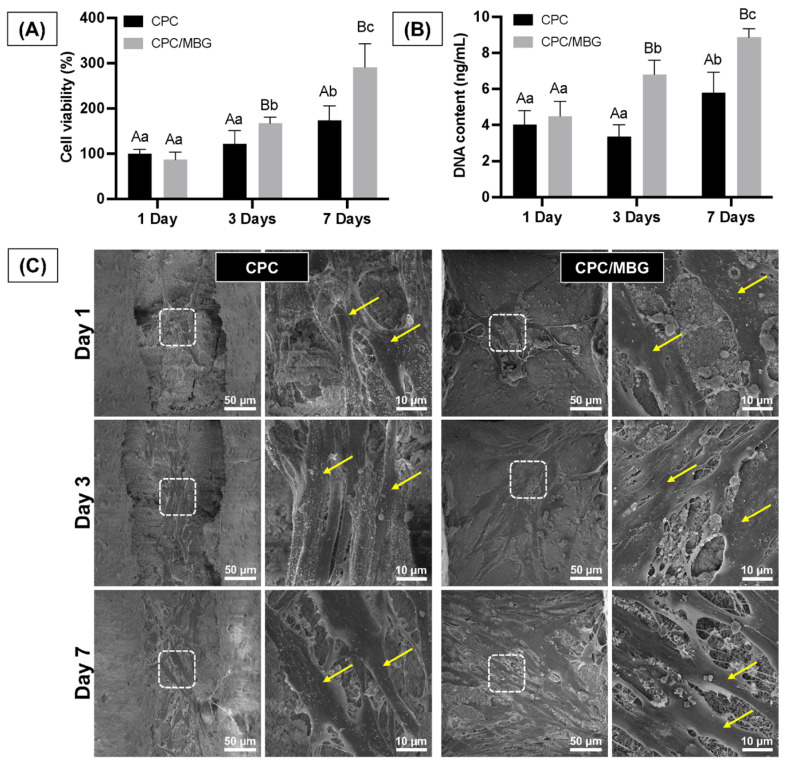
(**A**) Cell viability assessed by the alamarBlue assay and (**B**) DNA content quantified using the Quant-iT PicoGreen assay for alveolar bone-derived mesenchymal stem cells (aBMSCs) seeded onto the scaffolds after 1, 3, and 7 days. Mean values are represented by the columns, and standard deviations are represented by the error bars (*n* = 4). Significant differences between groups are distinguished by distinct capital letters. Different lowercase letters denote statistical differences between time-points within each group (*p* < 0.05). (**C**) Exemplary scanning electron microscopy (SEM) micrographs revealing the overall morphology, adhesion, and spreading of aBMSCs on the scaffolds (*n* = 2). Arrows indicate cell spread on the scaffold surface.

Assessing the cells’ adhesion and spreading on the scaffold surface is essential, as these processes are the initial stages of guided tissue regeneration, followed by proliferation for effective tissue formation [50]. The assessment of the scaffolds’ cytocompatibility showed that both scaffold formulations supported similar cell viability after 1 day of cell culture (*p* > 0.05). However, after 3 and 7 days, the scaffolds containing MBG (CPC/MBG) supported significantly higher cell viability when compared to the unmodified scaffolds (CPC) (*p* < 0.05) (Figure 8A). A similar trend was seen in the DNA content assay, which showed no significant difference in DNA content for both scaffold types after 1 day (*p* > 0.05), followed by a significant increase in DNA content in the CPC/MBG group compared to the control after 3 and 7 days (*p* < 0.05) (Figure 8B).

The qualitative results of cell morphology, adhesion, and spreading also support the quantitative findings of cell viability and DNA content over time. The scaffolds showed similar densities of elongated and spread cells on their surfaces after 1 day of cell culture. However, after 3 and 7 days, a higher cell density was observed for the CPC/MBG formulation compared to the control group (Figure 8C). An increase in cell viability, DNA content, and the density of adhered cells on the scaffold surface over time can be linked to an increase in cell proliferation [51,52].

Other studies have also shown improvements in these biological properties with the addition of different types of bioactive glasses to the scaffold matrix [53,54,55]. These effects are likely due to ion release from the scaffolds and surface changes caused by incorporating bioactive glasses in specific forms, especially the release of Si^4+^ ions from MBG, which has been linked to increased cell viability and osteogenic effects [53,56]. Both surface modifications and Si^4+^ release were observed in our study, supporting the biological findings.

**Figure 9:**
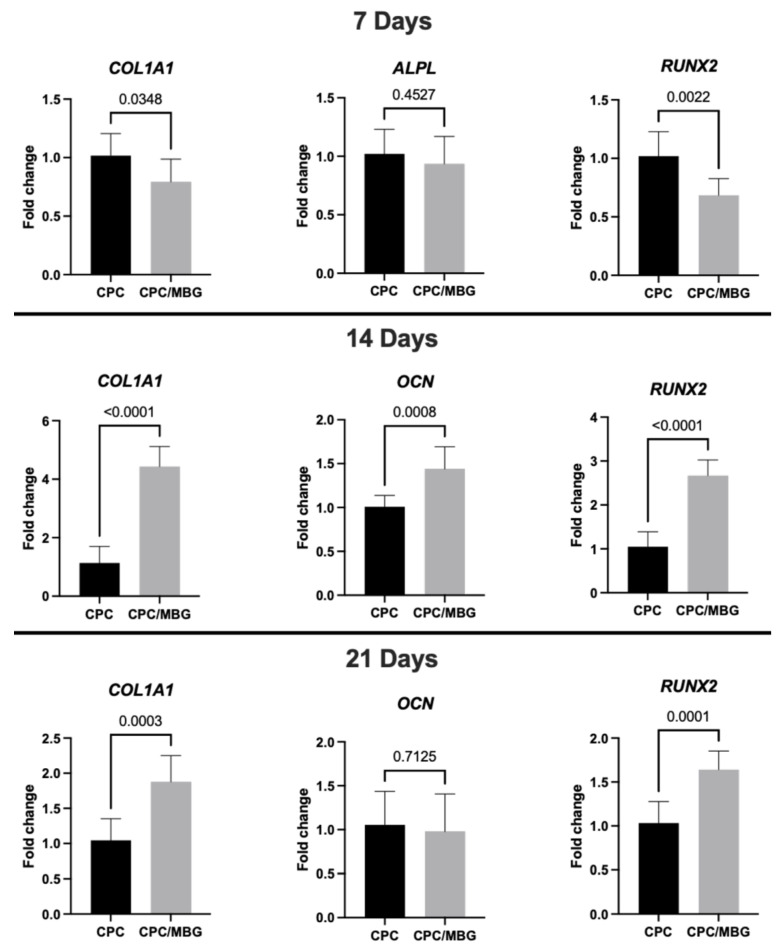
Gene expression (RT-qPCR) of *COL1A1*, *ALPL*, *RUNX2*, and *OCN* in alveolar bone-derived mesenchymal stem cells (aBMSCs) seeded on top of the scaffolds after 7, 14, and 21 days. Mean values are represented by the columns, and standard deviations are represented by the error bars, *n* = 4. (unpaired *t*-test, statistical difference for *p* < 0.05).

Regarding gene expression (Figure 9), after 7 days of cell culture, RT-qPCR analysis of osteogenic markers showed lower levels of collagen type I (*COL1A1*) and Runt-related transcription factor 2 (*RUNX2*) in the CPC/MBG group compared to the control group (CPC) (*p* < 0.05), although the expression levels of alkaline phosphatase (*ALPL*) were statistically similar between both scaffold formulations (*p* > 0.05). The initial downregulation of *COL1A1* and *RUNX2* in the CPC/MBG group may be attributed to an adaptation phase where cells respond to the modified scaffold surface chemistry and ion release profile. However, the similar *ALPL* expression suggests that early osteogenic commitment is not completely affected. Si^4+^ ions released from MBG can transiently alter early osteogenic signaling before enhancing differentiation at later stages by initially promoting cell proliferation, as observed for the cell viability and DNA content results. These ions have also been shown to stimulate cell proliferation of BMSCs and osteoblast-like cells through the activation of the ERK-MAPK pathway [57,58].

However, after the transient phase of increased cell growth, extracellular matrix accumulation, enhanced signaling, and changes in the cellular environment could trigger the upregulation of osteogenic markers, leading to a later stage of osteogenic differentiation. This was confirmed at 14 days, when the CPC/MBG scaffold formulation showed significantly higher expression levels of *COL1A1*, osteocalcin (*OCN*), and *RUNX2* compared to the control group (*p* < 0.05). The sustained release of Si^4+^ ions from MBG may have contributed to increased collagen production (*COL1A1*) and osteoblast maturation (*OCN*, *RUNX2*). It was previously demonstrated that the WNT and SHH signaling pathways could be involved in these processes [58]. Additionally, the rougher surface morphology observed via SEM may enhance cell–matrix interactions, contributing to the osteogenic gene expression [59]. The simultaneous increase in *RUNX2* (a master regulator of osteogenesis) and *OCN* (a late-stage marker) suggests that MBG incorporation can accelerate the transition from early to intermediate differentiation phases.

At 21 days, the expression of *COL1A1* and *RUNX2* increased significantly in CPC/MBG scaffolds compared to the control (*p* < 0.05). In contrast, *OCN* levels were similar between both scaffold formulations (*p* > 0.05) (Figure 9). The persistent upregulation of *COL1A1* and *RUNX2* confirms the long-term osteoinductive effect of MBG and ongoing extracellular matrix remodeling and osteogenic activity, which could translate to faster bone regeneration in vivo.

Taken together, our results showed that Si^4+^ ions released by MBG played an important role in the biological properties of CPC/MBG scaffolds, enhancing biocompatibility, promoting cell adhesion/spreading, and also upregulating important osteogenic gene expression. Furthermore, another remarkable feature that favors the biological activity is the greater surface area of MBG particles, conferred by its mesoporous structure [19]. The microchannels increase the surface area of MBGs, broaden the surface that is exposed to the environment, and consequently enhance the bioactivity of these particles [60].

Although our study yielded positive results, some limitations should be acknowledged. A thorough evaluation of bone regeneration requires additional assessments of osteoclast activity, angiogenic potential, and immune responses. Moreover, the lack of in vivo experiments limits the investigation of key aspects such as bone remodeling, inflammatory responses, and other host-related reactions. Another issue is the inability to add larger amounts of MBG into CPC due to limitations in the ink’s rheological properties and printability. Increasing the MBG content would necessitate adding more carrier liquid to the formulation [8], which was not feasible in this case because a commercial ink was used. Future studies should aim to enhance the carrier system and investigate the potential of CPC and MBG for other applications in bone tissue regeneration [5,61].

Furthermore, the degradation rate of the distinct scaffold formulations was not assessed in our study. CPC-based scaffolds are known to exhibit slow degradation in vivo [32,35]. Nonetheless, incorporating bioactive glasses, including MBG, into CPC has been reported to enhance its degradation ability [30,32,35].

## 4. Conclusions

The combination of CPC with MBG enhanced biological properties, including biocompatibility and osteogenic potential, as well as improved mechanical performance compared to CPC scaffolds. Therefore, CPC/MBG scaffolds are a promising alternative to standard CPC scaffolds for bone regeneration, and additional studies are recommended to further explore the potential of combining these materials.

## Data Availability

The original contributions presented in the study are included in the article, further inquiries can be directed to the corresponding author.
